# The Beekeeping State and Inventory of Mellifero-Medicinal Plants in the North-Central of Morocco

**DOI:** 10.1155/2021/9039726

**Published:** 2021-01-06

**Authors:** Meryem Bakour, Hassan Laaroussi, Nawal El menyiy, Tarik Elaraj, Asmae El ghouizi, Badiaa Lyoussi

**Affiliations:** Laboratory of Natural Substances, Pharmacology, Environment, Modeling, Health and Quality of Life (SNAMOPEQ), Faculty of Sciences Dhar El Mahraz, University Sidi Mohamed Ben Abdellah, Fez, Morocco

## Abstract

This study aims to determine the diversity of melliferous plants and to recognize the state of beekeeping in the Fez-Meknes region in Morocco. We conducted a questionnaire for beekeepers that set up their hives in the prefectures and provinces of the region, and we have studied the pharmacological evidence of the most preferred plants by beekeepers to assess its medicinal values. The results indicate that honey, bee pollen, bee bread, royal jelly, propolis, bee wax, bee venom, and bee queens are produced in this region with different percentages, and 102 plants belonging to 32 families were obtained in the inventory of melliferous plants; the most represented families were Asteraceae and Lamiaceae (13.73% each) followed by Rosaceae (8.82%). Among these 102 plants identified, 79 plants provide nectar and pollen for bees, 16 plants provide only pollen, 3 plants provide only nectar, 35 plants are resinous, and 6 plants provide honeydew for bees. The outcome of this study will contribute to the valuation of melliferous plants and help to establish a practical guide for the development of the beekeeping sector as an agricultural economic approach.

## 1. Introduction

Beekeeping is the art and science of breeding and caring for bees to exploit their products such as honey, bee pollen, bee bread, bee wax, bee venom, royal jelly, and the bee queens. It is a branch of agriculture that has been practiced for thousands of years by traditional methods, and it has evolved and modernized over the years [1]. Beekeeping is one of the essential pillars of agricultural development in Morocco, especially in the Fez-Meknes region where agriculture accounts for nearly half of the regional gross domestic product. The breed of bees that exists in Fez-Meknes is *Apis mellifera intermissa*; it is swarming with an aggressive behavior and is characterized by a good production of honey. This breed is famous for the overproduction of propolis, as well as enduring climate variations [2, 3]. In order to cover its nutritional and preventive needs, the bee collects substances including nectar, pollen, oil, and resin from plants that are highly attractive to them and also exudates of plant-sucking insects to produce honeydew where the hive products are reflected through the quantity and quality of the pollinated plants. These melliferous plants vary from one area to another depending on the biotic, climatic, and ecological factors [4].

Bees search for melliferous plants not only in forests, grasslands, ruderal, and marshy vegetation but also in agrophytocenoses, such as orchards, vineyards, rapeseed, sunflower, and alfalfa crops, or in medicinal plant plantations and aromatics [5]. By pollinating melliferous plants, bees play a vital role in achieving plant fertilization and in conserving biodiversity. As pollinators, bees are useful as samplers of the environment in which they are conserved and have been used as bioindicators of ecosystem's health. Bees in search of food can cover up to eight kilometers of radii for pollen, nectar, and resinous plant substances [6]. Encouraging beekeeping means strengthening agriculture and thus helping people to become less vulnerable to poverty [7]. To establish a detailed mapping of melliferous plants in Fez-Meknes, we conducted a questionnaire for professional beekeepers that set up their hives in the prefectures and provinces of the region. The objective of this study is to determine the richness of the Fez-Meknes region in melliferous plants and to highlight the state of beekeeping favoring the development and evolution of the bee sector.

## 2. Materials and Methods

### 2.1. Area of the Study

The region of Fez-Meknes is located in the northern center of Morocco (34° 02′00 ″north, 500′00″ west), integrating part of the Saïss plain alongside the mountain ranges of the Rif and the Middle Atlas, and it stretches over an area of 40.075 km^2^ representing 5.7% of the national territory. It is geographically limited by the Tangier-Tetouan-Al Hoceima region in the north, the Rabat-Sale-Kenitra region in the west, the Oriental region in the east, the Beni Mellal-Khenifra region in the southwest, and the region of Drâa-Tafilalet in the South [8].

**Figure 1:**
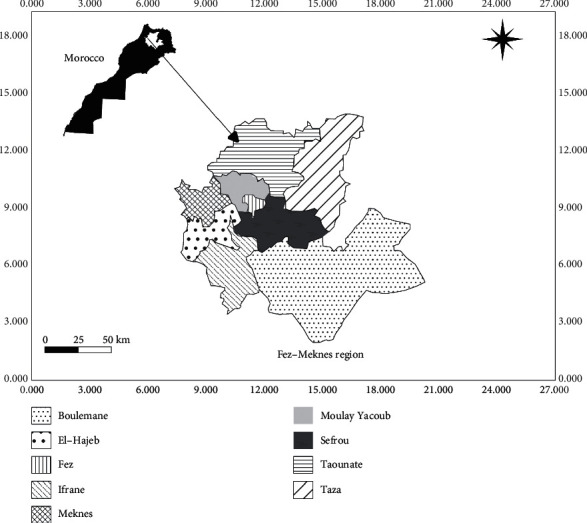
Map representation of area of the study.

The Fez-Meknes region includes nine prefectures and provinces: the prefecture of Fez, the prefecture of Meknes, the province of Boulemane, the province of Sefrou, the province of Moulay Yacoub, the province of Taounate, the province of Taza, the province of El Hajeb, and the province of Ifrane (Table 1 and Figure 1).

Based on the information given by the beekeepers and the data described elsewhere [8–10], the predominant vegetation in Fez is *Olea europaea* L var. sativa (cultivated), *Ceratonia siliqua* L (cultivated), *Capparis spinosa* L (cultivated), *Myrtus communis* L (native), and *Silybum marianum* (L.) Gaertn (native); the predominant vegetation in Meknes is *Olea europaea* L var. sativa (cultivated), *Capparis spinosa* L (cultivated), *Silybum marianum* (L.) Gaertn (native), *Mentha* spp. (cultivated), and *Ammi visnaga* (L.) Lam (native); the predominant vegetation in Boulemane is *Bupleurum spinosum* Gouan (native), *Peganum harmala* L (native), *Thymus vulgaris* L (native), and *Rosmarinus officinalis* L (native); the predominant vegetation in Sefrou is *Prunus cerasus* L (cultivated), *Prunus domestica* L (cultivated), *Ceratonia siliqua* L (cultivated), *Olea europaea* L var. sativa (cultivated), *Ruta graveolens* L (native), and *Quercus ilex* L (native); the predominant vegetation in Moulay Yacoub is *Capparis spinosa* L (cultivated), *Olea europaea* L var. sativa (cultivated), *Myrtus communis* L (native), *Ammi visnaga* (L.) Lam (native), *Silybum marianum* (L.) Gaertn (native), and *Agave sisalana* Perrine (cultivated); the predominant vegetation in Taounate is *Olea europaea* L var. sativa (cultivated), *Crataegus monogyna* Jacq (native), *Ficus carica* L (cultivated), *Ceratonia siliqua* L (cultivated), *Matricaria chamomilla* L (native), *Origanum vulgare* L (native), and *Prunus domestica* L (cultivated); the predominant vegetation in Taza is *Olea europaea* L var. sativa (cultivated), *Ceratonia siliqua* L (cultivated), *Arbutus unedo* L (native), *Tetraclinis articulata* (Vahl) Mast (native), *Quercus ilex* L (native), *Pinus halepensis* Mill (native), *Quercus suber* L (native), *Cedrus atlantica* (Manetti ex Endl) (native), *Rosmarinus officinalis* L (native), *Globularia alypum* L (native), *Ziziphus lotus* (L.) Lam (native), and *Dittrichia viscosa* (L.) Greuter (native); the predominant vegetation in El-Hajeb is *Ammi visnaga* (L.) Lam (native), *Myrtus communis* L (native), *Silybum marianum* (L.) Gaertn (native), *Thymus vulgaris* L (native), and *Crataegus monogyna* Jacq (native); and the predominant vegetation in Ifrane is *Thymus vulgaris* L (native), *Origanum vulgare* L (native), *Lavandula angustifolia* Mill (native), *Cedrus atlantica* (Manetti ex Endl) (native), and *Pinus halepensis* Mill (native).

### 2.2. Data Collection

In order to facilitate data collection for this study, 132 beekeepers were interviewed using a questionnaire focused on information about the beekeeper: gender, education level, age group, and duration of experience; information on the apiary: the number of hives, the amount of honey produced annually/hive, and other apicultural products provided by each beekeeper, as well as the prefecture or the province in the region Fez-Meknes preferred by the beekeeper for installing their apiary; and information on the honey plants that exist in each prefecture and province of the Fez-Meknes region: we asked beekeepers to give us a list of melliferous plants. Thus, we collected data on the information about the apicultural importance of each plant listed. The vernacular name of the plants was given by the beekeepers, and the scientific name was identified following Bellakhdar and Aafi et al. [11, 12].

Registration of all plant species observed by beekeepers was carried out throughout the year in the nine prefectures and provinces represented by the Fez-Meknes region. Each source has been identified by its vernacular name, scientific name, and botanical family. The average date of the flowering period of each plant and the apicultural value as a source of nectar and/or pollen, resin, and honeydew were recorded.

### 2.3. Data Analysis

Data were entered as codes in MS Windows Excel and then transferred to SPSS version 21 statistical analysis software.

## 3. Results and Discussion

### 3.1. Characterization of the Beekeeping Sector

#### 3.1.1. Information on Beekeepers and Their Apiaries

**Figure 2:**
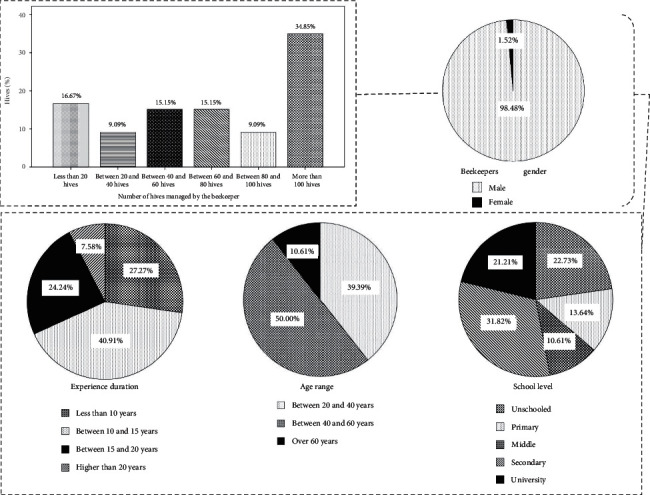
General information on beekeepers and their apiaries.

Beekeeping in Morocco has experienced a very important development in recent years, thanks to the contribution of the Morocco Green Plan [3]. Therefore, many people approach this activity with different motivations and interests. The results of this survey illustrated in Figure 2 show that all interviewees are professional beekeepers and beekeeping is their main source of economic income. The bee hive products especially honey is marketed through traditional circuits (localized souks in production areas and others local outlets, urban markets, and roadsides in the production areas) and modern circuits (supermarkets, hotels and tourist camps, and electronic services). In addition, the household income of the surveyed beekeepers increases positively during the International Exhibition of Agriculture organized annually in Meknes, which gives a valuable opportunity for the marketing of their products. Concerning the beekeepers gender, 98.48% is male and 1.52% is female, thus confirming a male predominance, which agrees with the results of Khabbach et al. [9] who showed that 90.9% of surveyed beekeepers in the pre-Rif region (Morocco) was male and only 9.1% was female, and this is because the beekeeping as a profession is relatively hard for women [13]. According to the opinion of the surveyed women in our study, the difficulty of beekeeping profession manifests mainly in the transhumance activity, which is done only at night and requires a high physical effort. The age range of the beekeepers questioned is the following: 50% of beekeepers are between 40 and 60 years old, 39.39% between 20 and 40 years, and 10.61% older than 60 years.

The school-level of the beekeepers surveyed is as follows: 31.82% have a secondary education level followed by 22.73% who are unschooled, 21.21% have a university education, 13.64% have a primary education, and 10.61% have a level of college education. These results affirm the importance of the academic and intellectual level in the apicultural practice, which requires a fundamental knowledge of melliferous flora and their relationship with environmental factors [14]. Concerning the years of experience in beekeeping, 40.91% of the beekeepers questioned have an experience between 10 and 15 years, 27.27% have an experience of less than 10 years, 24.24% of beekeepers have an experience between 15 and 20 years, and 7.58% of beekeepers have an experience more than 20 years. Concerning the number of hives in apiaries, 34.85% of the beekeepers surveyed manage apiaries made up of more than 100 hives, 16.67% have apiaries with fewer than 20 hives, 15.15% of beekeepers have apiaries made up of 40–60 hives, 15.15% of beekeepers have apiaries made up of 60–80 hives, 9.09% have between 80 and 100 hives, and 9.09% have an apiary made up of 20–40 hives. These results illustrate the region's potential and its contribution to the national honey production.

#### 3.1.2. The Preferred Locations by Beekeepers in the Fez-Meknes Region to Put Their Beehives

**Figure 3:**
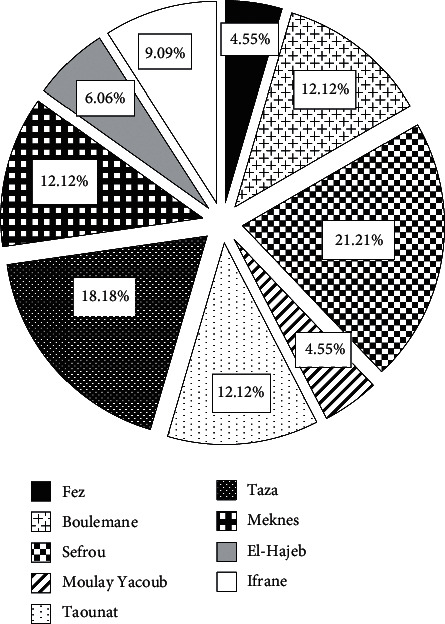
Distribution of beehive installation locations in the Fez-Meknes region.

Regarding the location preferred by the beekeeper for the installation of hives in the Fez-Meknes region (Figure 3), 21.21% of beekeepers prefers Sefrou, 18.18% prefers Taza 12.12% prefers Taounate, 12.12% prefers Boulemane, 12.12% prefers Meknes, 9.09% prefers Ifrane, 6.06% prefers El-Hajeb, 4.55% prefers Moulay Yacoub, and 4.55% prefers Fez. The choice of the installation area was based on the climatic characteristics, the type of vegetation, and the increased market demand for certain types of monofloral honey such as carob (*Ceratonia siliqua* L), arbutus (*Arbutus unedo* L), oregano (*Origanum vulgare* L), and jujubier (*Ziziphus lotus* (L.) Lam) honeys.

#### 3.1.3. Types of Bee Products Supplied by Beekeepers

**Figure 4:**
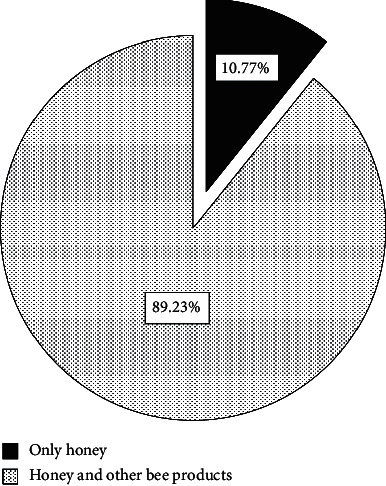
Percentages of honey and others bee hive production.

Concerning bee products supplied by beekeepers (Figure 4) and (Table 2), 10.77% of beekeepers produced and marketed only honey, while 89.23% produced and marketed honey and other bee products by the following percentages: 83.30% bee wax, 63.60% fresh bee pollen, 43.90% propolis, 22.70% dry bee pollen, 15.20% bee queens production, 7.60% royal jelly, 4.50% bee venom, 3% fresh bee bread, and 0% dry bee bread.

Among the famously used hive products, honey is the one that leads the list; hence, it is known that the beekeeping sector is essentially interested in the production and marketing of honey, in the second position is the bee wax, a very high percentage of beekeepers who have been surveyed (83.30%) produce bee wax, and the majority of the production is for hive recycling purposes, in the third place, we find the fresh bee pollen, followed by propolis in fourth. Since the surveyed population has a relatively high educational level, we observed that they are very well aware of the nutritional and therapeutic values of the other hive products such as bee pollen and especially fresh bee pollen, propolis, royal jelly, bee bread, and bee venom, but these last three products are poorly produced in the Fez-Meknes region and even throughout the Moroccan territory due to the difficulties encountered in the production and harvesting of these products. For instance, the royal jelly implies the breeding of the queens and requires precise techniques to keep its therapeutic value, which is very sensitive to temperatures above 4°C [15]; similarly, for bee venom, that requires specific devices introduced to the entrance of the hive that cause a weak electric field and stimulates the bees to release the venom on the device [16]. Furthermore, the fresh bee bread is rarely produced in the region; the percentage of production is only 3%, and this is because most beekeepers do not master correctly its harvesting technique without destroying a part of the hive while doing so [17]. Moreover, the pharmacological benefits of this product are recently discovered, and it is still unknown to the public [18–21].

#### 3.1.4. Annual Honey Production per Hive

**Figure 5:**
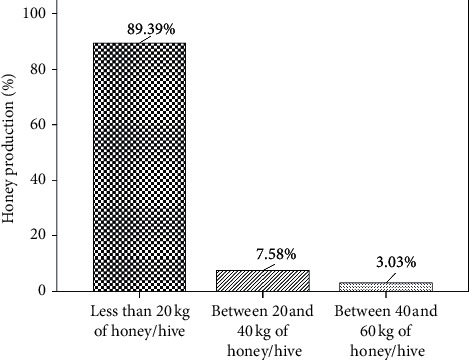
Annual honey production per hive.

Annual honey production per hive in Fez-Meknes is presented in Figure 5; the results indicate that 89.39% of apiaries produce less than 20 kg of honey per hive, 7.58% of apiaries produce between 20 kg and 40 kg of honey per hive, and 3.03% of apiaries produce between 40 kg and 60 kg of honey per hive. These findings show low honey production in the region; this may be due to *Varroa* mites' infection and bee poisoning by insecticides and climatic changes [22–24].

### 3.2. Taxonomic Diversity of Melliferous Plants in the Fez-Meknes Region

**Figure 6:**
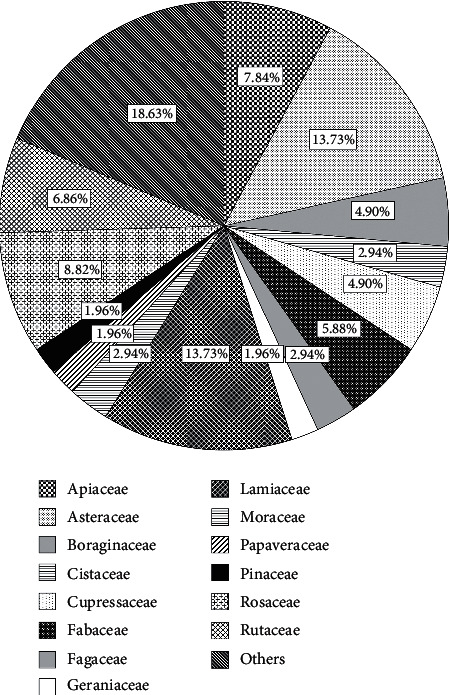
The most representative families of melliferous plants.

The information obtained by the beekeepers from the bee's foraging observations around the apiary made it possible to identify 102 melliferous plants belonging to 32 families (Figure 6). The most represented families in the established list of melliferous plants is the Asteraceae family with a percentage of 13.73% and Lamiaceae 13.73%, Rosaceae 8.82%, Apiaceae 7.84%, Rutaceae 6.86%, Fabaceae 5.88%, Cupressaceae 4.90%, Boraginaceae 4.90%, Cistaceae 2.94%, Fagaceae 2.94%, Moraceae 2.94%, Geraniaceae 1.96%, Papaveraceae 1.96%, and Pinaceae 1.96%. In a study published by Ennabili et al. (2000), the inventory of honey plants from the Mokrisset region (NW of Morocco) showed the presence of 78 species belonging to 35 families, and 48.7% of them corresponded to five families: Labiatae, Compositae, Rosaceae, Leguminosae, and Ericaceae [25]. In another study carried out in the central Rif region of Morocco, the most important taxa used by honeybees for nectar and/or pollen were *Ammi visnaga* (L.) Lam, *Mentha* spp.*, Eucalyptus camaldulensis* Dehnh., *Rubus ulmifolius* Schott, *Cannabis sativa* L, various grasses, and *Cistus* spp. [26].

Among the plants listed, we found 28 trees, 26 shrubs and undershrubs, 46 herbaceous plants, and 2 bulbs. 79 of these plants are classified as nectariferous and polliniferous; hence, they supply bees with pollen and nectar. 16 are polliniferous and they supply bees with pollen only, 3 are nectariferous and they supply bees with nectar only, 35 are resinous and they supply bees with resin, and 6 are honeydew plants and they supply bees with honeydew [14].

Sedentary beekeeping is the kind of beekeeping practiced by a traditional beekeeper or agri-beekeeper who leaves his hives in the same place and does not move them throughout the year. The bee's harvest radius in this type of beekeeping does not exceed 2-3 kilometers. This limits the production of the apiary. On the other hand, pastoral or transhumant beekeeping aims to move hives from site to site from 50 km to 500 km depending on the rate of flowering in melliferous regions [3]. The flowering period of the plants mentioned in the list (Table 3) varies according to the species; it spreads over the whole year from January until December. All beekeepers interviewees in this study are professional, and these conditions favor transhumance practices for them.

Regarding the type of melliferous plants preferred by beekeepers for the installation of hives (Table 4), 100% of beekeepers prefer to install their hives near nectariferous plants because they know the importance of this type of plants for honey production. In addition to nectariferous plants, 83.30% of beekeepers also prefer polliniferous plants, while 25.80% prefer resinous plants, and only 6% of beekeepers prefer honeydew plants. From these results, we can see that the beekeepers of Fez-Meknes know the importance of polliniferous and resinous plants to bees, considering the role of pollen and propolis for hives and bees' health, as well as for the marketing of these products in the region that are recently studied and consequently revealed to be rich in bioactive molecules with protective and therapeutic interest [10, 19, 27–29].

### 3.3. The Best Melliferous Plants and Their Medicinal Values

Regarding the opinion of beekeepers on the best vegetation that produce a good quality of propolis, most of them mentioned the following plants: *Pistacia lentiscus, Ferula communis, Populus alba, Pinus halepensis, Cedrus atlantica, Eucalyptus globulus, Ficus carica, Juniperus thurifera,* and *Tetraclinis articulata.* For the best nectariferous plants, the interviewed beekeepers cited the following plants: *Capparis spinosa, Calendula officinalis, Arbutus unedo, Ceratonia siliqua, Citrus* genus, *Bupleurum spinosum, Lavandula angustifolia, Lavandula multifida, Rosmarinus officinalis, Salvia officinalis,* and *Thymus vulgaris*. While for the good plants that provide the best pollen to bees, they have listed the following plants: *Capparis spinosa, Calendula officinalis, Prunus dulcis, Opuntia ficus-indica, Silybum marianum, Carthamus tinctorius, Centaurium erythraea,* and *Pelargonium odoratissimum.*

According to several studies, as shown in Table 5, the most preferred melliferous plants by beekeepers have a potent and large biological activities including antioxidant, antidiabetic, anticancer, antifungal, anti-inflammatory, and diuretic effects. It has been previously reported that the medicinal properties of bee products are highly dependent on many factors, including their botanical origins [74]; in fact, the diversity of their botanical sources gives them a wide range of pharmacological activities that can provide consumers a functional food product with therapeutic benefits.

## 4. Conclusion

The consensus of melliferous plants in the Fez-Meknes region made it possible to draw up an inventory of these plants and classify them according to the ingredients that they provide to the bee (nectar, pollen, resin, and honeydew) and the realization of a beekeeping calendar according to the flowering rhythms of each plant. The Fez-Meknes region offers great beekeeping potential, mainly due to the diversity of melliferous plants, allowing transhumance practices. An accompanying beekeeping policy is necessary to allow the optimal use of natural resources and a revitalization of this sector in order to improve the standards of living of the local population. This study contributes to the promotion of the field of beekeeping and to the valorization of the melliferous plants in the Fez-Meknes region.

## Figures and Tables

**Table 1:** Climatic and geographical information of the Fez-Meknes region.

Locality name	Latitude (N)	Longitude (W)	Altitude (m)	Pluviometry (mm)	Temperature (°C)
Fez	33°52′22.86″	5°32′26.63″	410	1–78	9.9 to 27.2
Meknes	33°52′22.86″	5°32′26.63″	546	2–93	9.8 to 25.9
Boulemane	33°21′46.3″	4°43′48.3″	1752	9–60	3.2 to 22.1
Sefrou	33°49′49.89″	4°50′7.14″	823	2.4–62.7	9.2 to 26.3
Moulay Yacoub	34°5′14.81″	5°10′42.25″	238	1–85	6 to 36
Taounate	34°32′12.9″	4°38′23.53″	600	1–101	9.1 to 26.5
Taza	34°12′38.02″	3°59′52.97″	550	2–91	9.5 to 28.2
El-Hajeb	33°41′8.65″	5°22′4.02″	1000	4–106	7 to 24.5
Ifrane	33°31′22.1″	5°6′39.44″	1664	8–122	2.7 to 21.8

**Table 2:** Types and percentages of bee hive production.

	Honey (%)	Fresh bee pollen (%)	Dry bee pollen (%)	Fresh bee bread (%)	Dry bee bread (%)	Royal jelly (%)	Propolis (%)	Bee wax (%)	Bee venom (%)	Production of bee queens (%)
The percentage of production	100	63.60	22.70	3	0	7.60	43.90	83.30	4.50	15.20

**Table 3:** List of melliferous plants in the Fez-Meknes region.

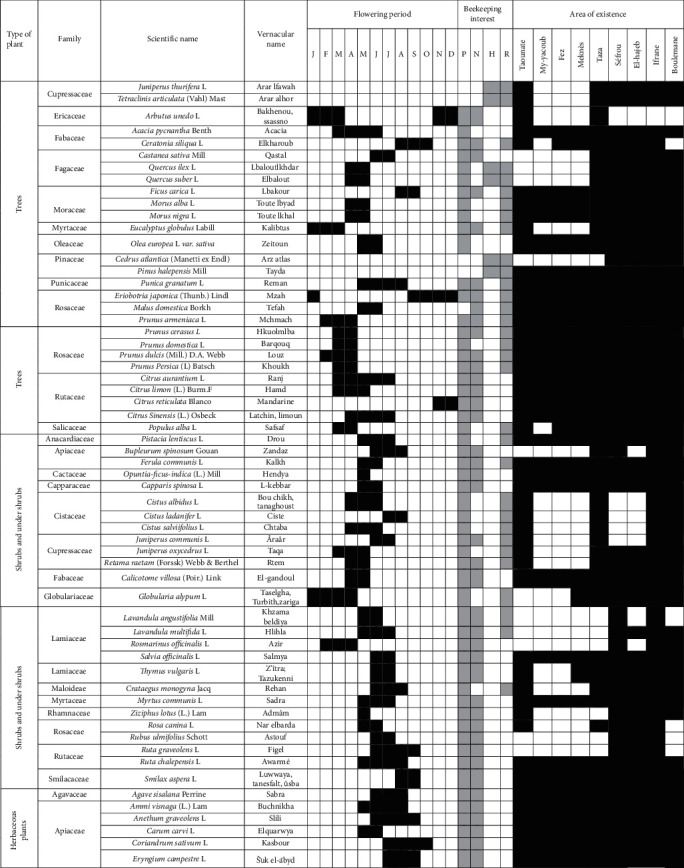
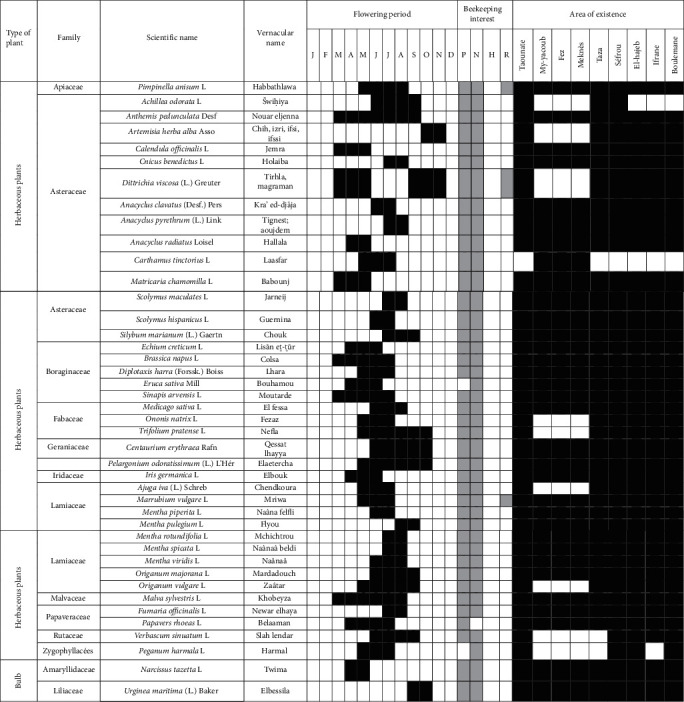

Flowering period: *J*, January; *F*, February; *M*, March; *A*, April; *M*, May; *J*, June; *J*, July; *S*, September; *N*, November; *D*, December. Beekeeping interest: *P*, pollen; *N*, nectar; *H*, honeydew; *R*, resin.

**Table 4:** Type of plants preferred by beekeepers for the installation of hives.

Type of plants preferred	Nectariferous plants (%)	Polliniferous plants (%)	Resinous plants (%)	Honeydew plants (%)
Percentage	100	83.30	25.80	6

**Table 5:** Review of the pharmacological evidence of the best melliferous plants existed in the Fez-Meknes region according to the beekeeper's opinion.

Type of plant	Scientific name	Pharmacological evidence
Resinous plants	*Cedrus atlantica* (Manetti ex Endl)	Antimicrobial effect [30]
	*Ferula communis* L	Antimicrobial and anticancer effects [31, 32]
	*Ficus carica* L	Antioxidant, antimicrobial, and anticancer effects [33, 34]
	*Eucalyptus globulus* Labill	Antioxidant, antifungal, and antimicrobial effects [35, 36]
	*Juniperus thurifera* L	Antioxidant and antibacterial effects [37, 38]
	*Pinus halepensis* Mill	Antioxidant and antibacterial effects [39, 40]
	*Pistacia lentiscus* L	Antioxidant, antidiabetic, and hepatoprotective effects [41, 42]
	*Populus alba* L	Antioxidant effect [43, 44]
	*Tetraclinis articulata*(Vahl) Mast	Antioxidant, antibacterial, anti-inflammatory, and cytotoxic effects [45–47]
Nectariferous plants	*Arbutus unedo* L	Antioxidant, antibacterial, anti-inflammatory, and antiaggregating effects [48–50]
	*Bupleurum spinosum* Gouan	Antioxidant effect [51]
	*Calendula officinalis* L	Healing and antimicrobial effects [52, 53]
	*Capparis spinosa* L	Antioxidant, hepatoprotective, and antidiabetic effects [54, 55]
	*Ceratonia siliqua* L	Antigenotoxic, antioxidant, antibacterial, and anticancer effects [34, 56, 57]
	*Citrus* spp	Antioxidant, vasodilator, antimicrobial, and antimutagen effects [58–61]
	*Lavandula angustifolia* Mill	Antioxidant effect [62]
	*Lavandula multifida* L	Anti-inflammatory effect [63]
	*Rosmarinus officinalis* L	Antihyperglycemic, antihyperlipidemic, and antioxidant effects [64]
	*Salvia officinalis* L	Antiproliferative effect [65]
	*Thymus vulgaris* L	Antioxidant and anti-inflammatory effects [66]
Polleniferous plants	*Carthamus tinctorius* L	Antioxidant effect [67]
	*Centaurium erythraea* Rafn	Antioxidant and antimicrobial effects [68]
	*Opuntia-ficus-indica* (L.) Mill	Diuretic, antioxidant, and anti-inflammatory effects [69, 70]
	*Pelargonium odoratissimum* (L.) L'Hér	Antifungal effect [71]
	*Prunus dulcis* (Mill.) D.A. Webb	Antioxidant, antiproliferative, antibacterial, and antidiabetic effects [72, 73]

## Data Availability

The data used to support the findings of this study are available from the corresponding author upon request.
